# Investigation of a Measles Outbreak in China to Identify Gaps in Vaccination Coverage, Routes of Transmission, and Interventions

**DOI:** 10.1371/journal.pone.0133983

**Published:** 2015-07-24

**Authors:** Xiang Zheng, Ningjing Zhang, Xiaoshu Zhang, Lixin Hao, Qiru Su, Haijun Wang, Kongyan Meng, Binglin Zhang, Jianfeng Liu, Huaqing Wang, Huiming Luo, Li Li, Hui Li, Chao Ma

**Affiliations:** 1 National Immunization Program, Chinese Center for Disease Control and Prevention, Beijing, China; 2 Chinese Field Epidemiology Training Program, Chinese Center for Disease Control and Prevention, Beijing, China; 3 Taizhou Prefecture Center for Disease Control and Prevention, Taizhou Prefecture, Zhejiang Province, China; 4 Gansu Provincial Center for Disease Control and Prevention, Lanzhou, Gansu province, China; 5 Longnan Prefecture Center for Disease Control and Prevention, Longnan Prefecture, Gansu Province, China; 6 Wudu County Center for Disease Control and Prevention, Longnan Prefecture, Gansu Province, China; University of Florida, UNITED STATES

## Abstract

**Background:**

A measles outbreak occurred in a western county of China in 2013, the year after China’s historic nadir of measles. We conducted a field investigation to identify gaps in measles vaccination coverage and immunization program weaknesses, and to provide recommendations for measles outbreak response and immunization program improvement.

**Methods:**

We analyzed surveillance data from 2008 to 2013 to describe the measles epidemiology of the county. Measles-containing vaccine coverage was estimated using two methods: previously-reported administrative coverage and an estimation of coverage by clinic-kept vaccination records (n = 542). We conducted a rapid field coverage assessment in a migrant population village to evaluate coverage after emergency vaccination. We conducted a review of hospital records of measles cases to address the role hospital transmission played during the early stage of this outbreak.

**Results:**

There were 153 cases in the outbreak, primarily among children too young to vaccinate, unvaccinated children less than 3 years old, and adults. Measles-containing vaccine coverage by the field assessment showed that 20% of children aged 8–17 months had zero doses, and 9% of ≥2 years old children had fewer than two doses. The vaccination statuses of all adult cases were either zero doses or unknown. At least 61% of cases had been hospitalized. The proportion of cases who had been hospital-exposed 7 to 21 days prior to rash onset decreased from 52% to 22% after hospitals strengthen their isolation measures.

**Conclusions:**

This outbreak was a result of measles vaccination coverage gaps among young children and adults, and insufficient hospital isolation of cases. The lower coverage seen in the field estimation compared with reported coverage showed that reported coverage could have been overestimated. Hospitals were sites of transmission in the early stage of the outbreak. A strict hospital isolation policy could decrease spread of measles. Emergency vaccination was associated with stopping measles transmission in low coverage areas.

## Introduction

Measles is a highly contagious infectious disease that can be eliminated by immunization program strategies that are guided by laboratory-supported surveillance. China has been implementing a routine, two-dose schedule of measles containing vaccine (MCV), with one dose of measles and rubella vaccine given at 8 months of age, and one dose of measles, rubella, and mumps vaccine given between 18 months and 24 months of age [1]. As China works toward the elimination of measles, outbreaks of the indigenous measles genotype still occur. These outbreaks offer opportunities to identify program weaknesses that can be improved and provide opportunities to evaluate outbreak response efforts.

In March 2013, a measles outbreak occurred in Wudu County (W county) in western China’s Gansu province. During May 3^rd^ to 8^th^, the National Immunization Program of the Chinese Center for Disease Control and Prevention (China CDC) was asked by Gansu province CDC to support their ongoing investigation and response to this outbreak. We report the results of our efforts to identify causes of this outbreak and key strategies for emergency response.

## Methods

### Setting

W county has 560 thousand residents and a population density of 124 people per square kilometer. Routine immunization services are offered at no charge to parents. Each of the 36 townships in W county has one large clinic that provides vaccination services. Data from China’s National Notifiable Disease Reporting System (NNDRS) showed that in 2008 the county had a peak measles incidence of 68.5 cases per 100,000 population. The age distribution of cases in 2008 was: 12% were less than 8 months old; 10% were between 8 months and 17 months old; 15% were between 18 months and 3 years old; 35% were between 4 years and 17 years old; and 28% were over 17 years of age.

Between 2008 and 2012, W county reported 2-dose, routine MCV administrative coverage to be greater than 95% each year. Since 2008, W county has performed four rounds of supplementary measles immunization activities (SIA) in addition to the 2-dose routine immunization policy (Table 1). Reported administrative coverage for each SIA was greater than 95%, which is similar to reported coverage from SIAs elsewhere of China [2–4].

**Table 1:** Supplementary immunization activities (SIAs) and screening-and-vaccination activities (SVA) conducted during 2008–2013, W county, China.

Time	Category	Age range	No of vaccinated	Total	Coverage %(95% CI)
2008 †	SIA	8 months-14 years	87041	90275	96.4 (96.3–96.5)
September 2010 ‡	SIA	8 months-4 years	27069	27268	99.3 (99.2–99.4)
September 2011	SIA	8 months-14 years	89731	92722	96.8 (96.7–96.9)
March to April 2012	SVA	8 months-6 years	5737	5800	98.9 (98.6–99.2)
March 2013	SIA	8 months-4 years	33852	34984	96.8 (96.6–96.9)

† An SIA conducted after the Wen-chuan great earthquake.

‡ As part of China’s nation-wide SIA in 2010

### Case data collection

We analyzed NNDRS measles surveillance data from Jan 1^st^ to the end of July 2013 to determine the basic epidemiology of the outbreak. According to the Chinese National Measles Surveillance guidelines [5], measles is required to be reported to China’s Internet-based NNDRS, and county CDC staffs are responsible for infectious disease case investigations and collection and transportation of blood specimens. A suspected case was defined as a person presenting with fever, rash, and one or more of cough, coryza, or conjunctivitis; a laboratory-confirmed case was a suspected case with a positive measles IgM antibody test or measles virus isolation result; and a clinically-diagnosed case was a suspected case without positive laboratory evidence of infection, but with either epidemiological linkage to a laboratory-confirmed case or without a clear diagnosis other than measles. In this study, we analyzed data from laboratory-confirmed and clinically-diagnosed measles cases. Consistent with Chinese National Measles Surveillance guidelines, we defined the incubation period of measles as 7–21 days before the rash onset date. Hospital-exposed means a person who had visited any hospital at least once, regardless of the reason for the visit.

The measles vaccination history for each case was determined through record review, and was categorized into one of five types when reported to NNDRS: zero doses, one dose, two doses or more, unknown, and missing dose information. For our analyses, we combined unknown and missing into a single category. All data were analyzed with the use of Microsoft Office Excel 2010 software.

### Identifying coverage gaps

In addition to examining reported administrative MCV coverage from routine immunization, SIAs, and screening and vaccination activities (SVA), we estimated clinic-based MCV coverage by review of clinic-kept vaccination records. Reported administrative MCV coverage had been determined by dividing the number of clinic-administered doses by the number of children registered in the clinic.

SIAs and SVAs were organized by the county government and conducted by the clinics. SIA coverage was determined by dividing the number of clinic-administered doses, regardless of individual’s MCV history, by the size of the clinic-reported target population of each SIA. SVA coverage was determined by dividing the number of clinic-administered doses by the size of the clinic-reported target population who had not yet received the full MCV regimen for their age group (8 months to 17 months of age children should have received one MCV dose and children over 18 months of age should have received 2 doses). The clinic-reported target populations included both locally-registered children and non-locally-registered children. Individuals with contraindications to measles vaccine at the time of vaccination were excluded from the denominator. The county government requested that clinics find and record information on all children living in their jurisdiction.

We reviewed vaccination records of children born between Jan 1, 2008 and July 15, 2012 from the five clinics of five townships (there is only one vaccination clinic in every township) with the most measles cases (accounting for 36% of all cases reported before review) in order to estimate measles vaccination coverage and on-time vaccination in these townships. We transcribed records from a random sample of 20 to 25 children per birth cohort per township clinic, for a total of 542 children.

### Review of hospital records of measles cases

By analyzing surveillance data from when the first cases were reported until the end of April, we found that approximately 53% cases had been hospital-exposed during their incubation periods. We conducted a review of hospital records of all measles cases reported by any of 3 hospitals reporting the most cases in this outbreak. An attempt was made to identify all admission and discharge dates from these cases between February 1 and May 5.

### Rapid field coverage assessment

We found a clustering of cases in a suburban village in which many migrant families from different townships lived. We conducted a rapid coverage assessment targeting children aged 8 months to 14 years of age using house-to-house visits in the morning, before the start of school. We divided investigators into two groups, each led by an EPI officer, and walked in an opposite directions to identify for 20 children for each group. Once an age-eligible child was found, we asked parents for the child’s vaccination certificates. We found 39 children, but 4 parents of 4 children had left vaccination certificates in their hometowns, leaving a total of 35 records available for review.

### Ethical considerations

This study did not involve endangered or protected species, and no human subjects were obtained. Administrative (doses administered) data, coverage survey data, and vaccination record review data that were collected as part of a vaccine preventable disease outbreak investigation are considered by China CDC’s Ethical Review Committee to be exempt from IRB review. Therefore, informed consent was not obtained for accessing administrative, survey, and immunization clinic record data. Individual identifying data were not retained in analytic data sets.

## Results

### Outbreak profile

**Fig 1:**
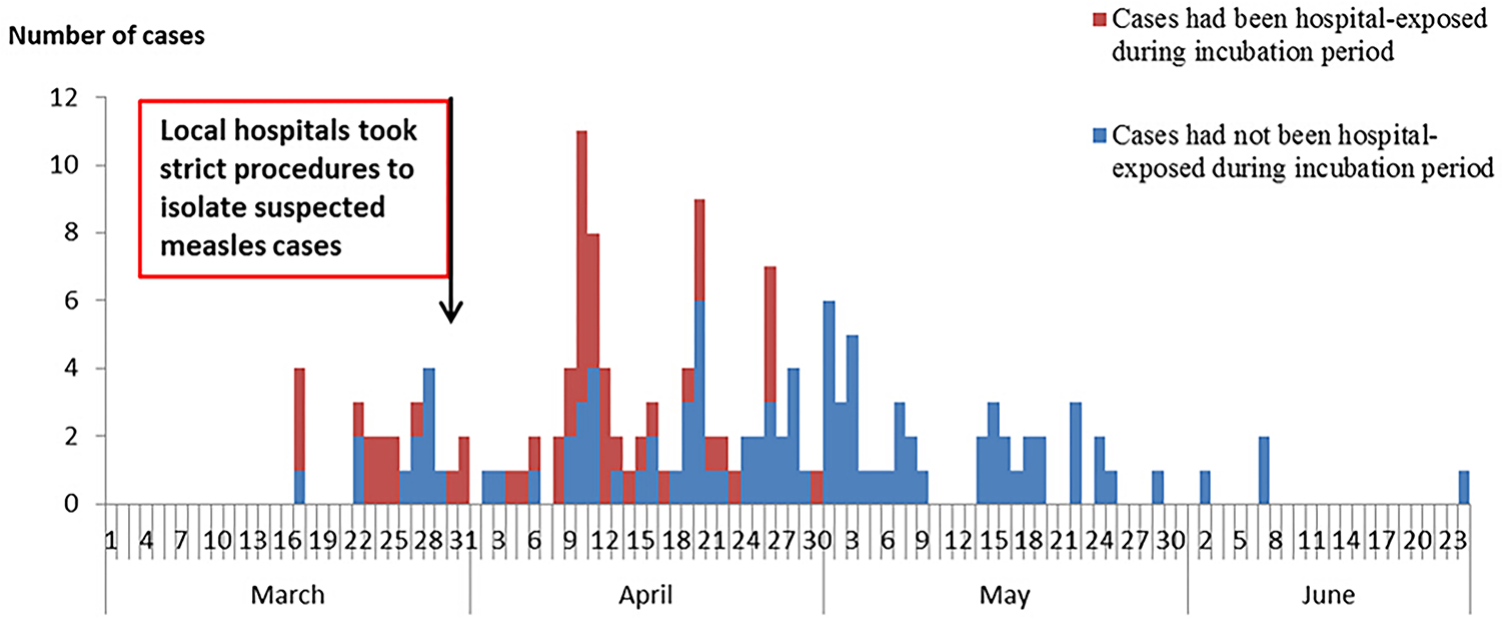
Time distribution of measles cases by illness onset date, categorized by hospital exposure history.

**Fig 2:**
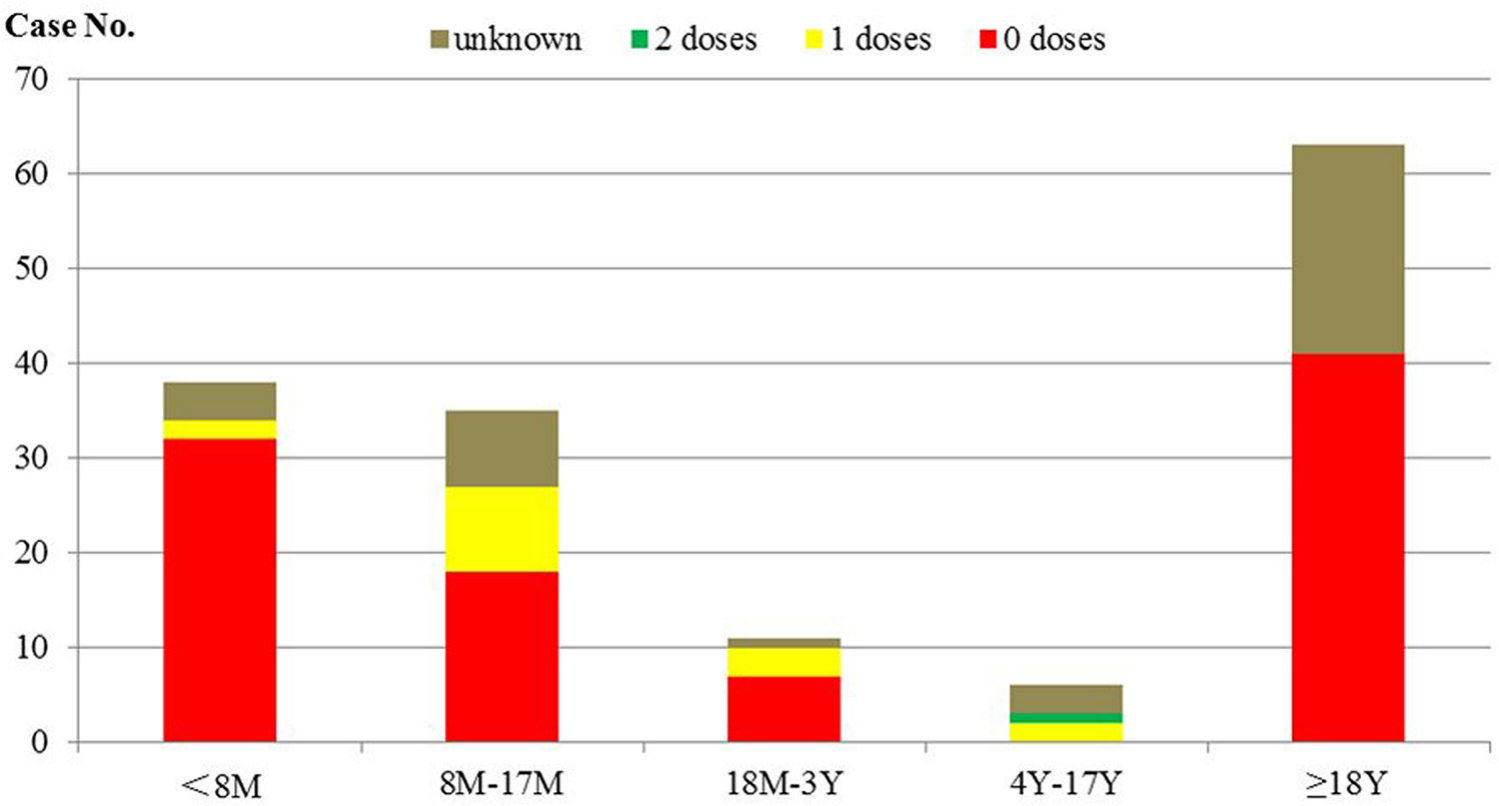
Distribution of MCV vaccination status among different age groups.

W county reported 153 confirmed cases (8% clinically diagnosed, 92% laboratory-confirmed) out of 165 suspected cases. The first case was reported on March 21^th^ (illness onset date was March 17^th^), and the last cases were reported in June (Fig 1). The ratio of males to females was 1.25:1, and the incidence rates were 29.1 /100,000 and 25.9/100,000, respectively. The overall incidence rate of 27.6/100,000 for this outbreak was higher than that of any corresponding period from 2009 to 2012, which were 7.9, 5, 3.5, and 0 per 100,000, respectively. The number of cases increased rapidly to a peak at the third week (April 8^th^ to 14^th^) after the first case became ill. By residence, cases were scattered in 30 of the 36 townships in W County. Among the cases, 25% were less than 8 months old; 23% were between 8 months and 17 months old; 7% were between 18 months and 3 years old; 4% were between 4 years and 17 years; and 41% were more than 17 years old. Among all of the cases aged more than 7 months, 13% had received at least one MCV dose. Among cases between 8 months and 17 months old, 26% had received one MCV dose. Among the 63 adults, the vaccination status was either 0 doses or unknown (Fig 2).

### Estimation of clinic MCV coverage

The clinic record review showed that 20% of children, aged 8 months to 17 months, had zero MCV doses; 39% children aged between 18 months and 23 months had one MCV dose or no dose; 91% of children ≥24 months of age had at least two doses [Table 2]; 56% of children had received their first MCV dose (MCV1) at 8 months of age[Table 3]; 62% of children had received their second MCV dose (MCV2) at 18–24 months of age; and 15% of children had received their second dose between 9–17 months of age [Table 4].

**Table 2:** MCV coverage assessment of different age groups*.

Age range	0 dose		1 dose		≥2 doses	
	No.	Percentage (95% CI)	No.	Percentage (95% CI)	No.	Percentage (95% CI)
8–17 months	22	19.6 (12.7–28.2)	62	55.4 (45.7–64.8)	28	25.0 (17.3–34.1)
18–23 months	2	3.5 (0.4–12.1)	20	35.1 (22.9–48.9)	35	61.4 (47.6–74)
≥24 months	7	1.9 (0.8–3.8)	25	6.7 (4.4–9.7)	341	91.4 (88.1–94.1)
sum	31	5.7 (3.9–8)	107	19.7 (16.5–23.3)	404	74.5 (70.7–78.2)

*The MCV doses include both routine immunization doses and SIA/SVA doses.

**Table 3:** Distribution of MCV1 vaccination month

Vaccination month	Number of records	%
8	320	59.0
9	85	15.7
10	32	5.9
11	29	5.4
12	11	2.0
13	7	1.3
14	2	0.4
15	3	0.6
16	3	0.6
17	3	0.6
≥18	16	3.0
unvaccinated	31	5.7
Total	542	100

**Table 4:** Distribution of MCV2 vaccination month.

Vaccination month	Number of records	%
9–17	62	14.8
18	94	22.5
19	58	13.9
20	30	7.2
21	28	6.7
22	21	5.0
23	21	5.0
24	9	2.2
≥25months	53	12.7
unvaccinated	42	10.0
Total	418	100

### Hospitalization information of measles cases

**Fig 3:**
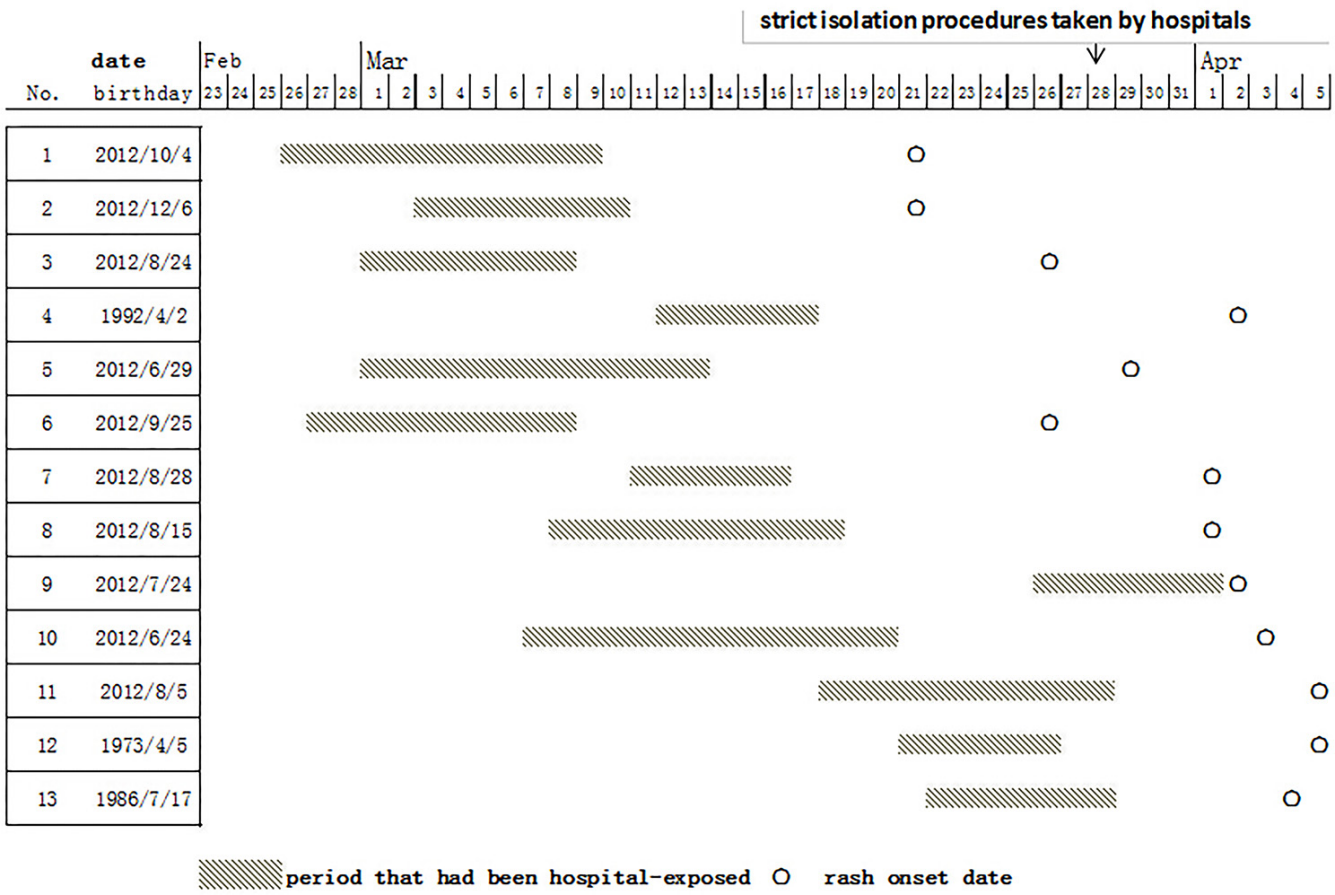
Hospital exposure histories prior to rash onset for 13 cases.

The field review showed that among the 94 cases hospitalized (61% of the total), 52 had been hospitalized after the onset of measles, and 42 had been hospital-exposed during their measles incubation period. There were 25 cases whose illness onset dates were before April; 13 of these 25 cases (52%) had been hospital-exposed during their incubation period; 10 of the 13 cases (77%) were less than one year old (Fig 3). All of the 13 cases had been hospital-exposed before or after 4 days of their rash onset. The sources of infections could not be identified. Age stratified data showed that 35% of cases (54/153) had been hospital-exposed during their incubation period and 44% of cases (20/45) were between 8 months and 2 years old.

### Transmission linked to hospitalized cases from a migrant village

There were 11 cases that resided in and around a densely populated village located in a suburb of W County. In this village, approximately 60% of residents are from other townships. This village does not offer routine immunization services to children who are not registered in the village due to limited public health resources. Nine out of the 11 cases in this village were among migrant people, but only one case had a vaccination record. We were able to contact 6 cases, and among these, 3 had been exposed to an index case who had been hospitalized for treatment but who returned to the village while still ill, and 2 had been hospitalized between 1 and 3 weeks prior to their rash onset.

### Emergency public health responses

The local government took several measures in response to the outbreak. Starting March 28^th^, the local hospitals implemented strict isolation measures, including using set-aside rooms to receive suspected measles cases, and widespread distribution of masks for respiratory precautions. Surveillance was intensified, as the government implemented a daily reporting system for all health care institutions to detect and confirm suspected cases as soon as possible. Close contacts were notified to monitor their temperature for 21 days after their last contact with a measles case, and to report to community doctors if fever or rash appeared.

On April 12^th^, a countywide emergency MCV vaccination campaign was initiated. The campaign included screening and vaccination activities in some townships and unselective SIAs in other townships. The target ages included children 8 months to 14 years of age. Each township conducted their campaign before April 25^th^. A rapid, countywide coverage assessment was conducted from April 26^th^ to 28^th^.

### Impact of responses

Between March 17^th^ and March 28^th^, 52% of cases had been hospital-exposed during their measles incubation period. On March 28^th^, measures to strengthen isolation in hospitals were implemented. After fourteen days following implementation of isolation measures, the proportion of cases that were hospital-exposed during the incubation period decreased to 22% (21 hospital-exposed cases / (97 total cases from April 12^th^ and after). Of 46 cases occurring after April, none were hospital-exposed during the incubation period. (Fig 1)

The emergency MCV immunization campaign that started on April 12^th^ vaccinated 40,231 children with single-antigen measles vaccine. The follow-up countywide coverage assessment showed: one-dose MCV coverage of children between 8 months and 18 months was 94.9%; two-dose MCV coverage of children between 18 months and 2 years was 95.9%; and two-dose MCV coverage of children between 18 months and 6 years was 97.6%.

Prior to the April immunization campaign, a rapid coverage assessment conducted by Gansu CDC in the migrant village (mentioned above) showed that 2-dose MCV coverage among children 18 months to 4 years of age was 48% (11/23). After the emergency vaccination campaign, the assessed MCV coverage with one or more doses was 100% among 35 children. Two-dose MCV coverage among children aged 18 months to 4 years increased from 48% of pre-campaign to 87% (13/15). Two-dose MCV vaccination coverage among children aged 18 months to 14 years was 88% (21/24). Eleven percent of children assessed had received their first-ever measles vaccine in this campaign. There were no measles cases reported from this village after April 22^nd^.

## Discussion

This measles outbreak of 153 cases was primarily among young children and adults, lasted for 10 weeks, and ended after an 8-week response that included enhancing surveillance, assessing coverage, improving hospital measles precautions, and emergency vaccination. Hospitals were shown to be significant points of transmission to a community with a routine immunization program that did not include non-registered children. Our investigation showed differences between official administrative coverage assessments and survey-based coverage assessments, with survey-based coverage being lower.

### Interpretation of findings

This outbreak appears to have been triggered by transmission of measles from one or more hospitals to villages with low vaccination coverage. Local hospitals lacked measles isolation measures, likely facilitating transmission. Once the hospitals changed their management of suspected measles cases, fewer children acquired measles in the hospitals.

Nearly one third of cases were among children 8 months to 3 years of age, and the majority of these children had not received measles vaccination. Our coverage assessments showed lower-than-reported coverage and a lack of on-time vaccination, since fewer than 60% of children received MCV1 during their 8^th^ month of life, and only 77% of children received their MCV2 before 24 months of age. Thus, the outbreak happened as susceptibles had accumulated. W county reported that vaccination services for 84% of the county population were provided on fewer than 5 days per month.

The age distribution of measles cases changed after several SIAs have targeted young children since 2008. The changing age distribution showed that the SIAs had some impact, but, nevertheless, this outbreak still occurred. This may be because SIAs are periodic [6] and may not cover all children as per government requirement. Unselective SIAs result in some too-early vaccinations and many repeated vaccinations, and increase risk for adverse events following immunization. Although we could not assess coverage of adults, the presence of either-zero-or-unknown vaccination records of adults, coupled with many cases of measles among children too young to vaccinate, raises challenges for China’s elimination of measles.

Delayed vaccinations are not rare in China or other countries. Timely vaccination with high coverage is critically important for measles elimination. Our estimate of coverage during this outbreak was lower than the 95% that had been reported based on administrative data. A field outbreak investigation that was conducted in 2013 in Hunan province of China also reveals a similar discrepancy [7].

Transmission of measles in health care facilities is well-known and has been also reported in many different settings [8–11]. An investigation in the Republic of Korea showed that nosocomial transmission appeared to precede community transmission when nosocomial and community transmission of measles coexisted in the same outbreak [8]. This phenomenon also was seen in our investigation. It is possible that unidentified measles cases occurred in the community and in hospitals prior to the onset of the outbreak [8]. Lack of ventilation in some hospitals may facilitate spread of measles virus [12], as can a high density of patients in a crowded healthcare facility [13, 14]. Strict isolation procedures were temporally associated with reduced transmission of measles in this outbreak as has been seen in other outbreaks in China [13]. Nosocomial transmission of measles primarily affects children [14, 15]. In our investigation, the distribution of cases hospitalized at early stage of the outbreak showed the susceptibility of young children.

The risk brought by low immunization coverage among migrants seen in this investigation has also been seen in other parts of China [2, 16, 17] and in other developing countries [18–20]. According to China’s Sixth Population Census in 2010, there were more than 261 million migrants in China, which was an increase of 81% over China’s Fifth Population Census in 2000 [21].

### Recommendations

This investigation and response support some recommendations. (1) Health authorities should immediately notify all hospitals in their jurisdiction if measles is identified in their communities so that the hospitals can review and strengthen their measles management and isolation procedures. (2) Measles patients who are able to be cared for and isolated at home or in their community should not be hospitalized. (3) Routine immunization services should be offered to all children living in a community, not just children registered in the community. (4) Vaccination services should be offered frequently enough to provide timely vaccination access. (5) Research and evaluation should be conducted to determine how to reduce the incidence of measles among adults. (6) Administrative coverage assessment methods should be augmented with survey-based coverage assessments to identify areas with low vaccination coverage. (7) Well-conducted screening and vaccination activities should be considered as alternatives to nonselective SIAs.

### Limitations

There are some limitations in this investigation. First, the estimation of MCV coverage was based on records from clinics in the five townships that had the largest number of cases. Thus, our coverage estimate cannot be generalized to the entire county. Second, although vaccination data is supposed to be transcribed from vaccination certificate to the clinic’s record shortly after vaccination, delays can happen, which may lead to a slight underestimate of coverage. Third, this investigation and response was not a controlled trial, and so the impact of the interventions was inferred only by temporal association. Fourth, some data were obtained from various reporting and recording systems (e.g., NNDRS, case investigation forms, and hospital medical records), which may be incomplete. Fifth, coverage levels were used as a proxy for immunity, which can be better assessed with serological surveys.

## Conclusions

This outbreak provided an opportunity to study routes of transmission and program weaknesses in order to not only stop the outbreak, but also to learn lessons that can be valuable in the China immunization program’s effort to eliminate measles. Improving the routine program efforts to measure coverage, include all children in the jurisdiction, conduct effective campaigns, and reduce nosocomial measles transmission has implications for other parts of China and other countries.

## Supporting Information

S1 Dataset Dataset of 153 cases. (XLSX)
