# Correction: Investigation of a Measles Outbreak in China to Identify Gaps in Vaccination Coverage, Routes of Transmission, and Interventions

**DOI:** 10.1371/journal.pone.0168222

**Published:** 2016-12-09

**Authors:** Xiang Zheng, Ningjing Zhang, Xiaoshu Zhang, Lixin Hao, Qiru Su, Haijun Wang, Kongyan Meng, Binglin Zhang, Jianfeng Liu, Huaqing Wang, Huiming Luo, Li Li, Hui Li, Chao Ma

## Notice of Republication

This article was republished on September 1, 2016, as the original S1 Dataset was published in error. Please download this article again to view the correct version.
